# Editorial: Factors contributing to dopaminergic cell death

**DOI:** 10.3389/fnmol.2023.1136930

**Published:** 2023-01-17

**Authors:** Sandra Blaess, Antonio J. Herrera

**Affiliations:** ^1^Neurodevelopmental Genetics, Institute of Reconstructive Neurobiology, Medical Faculty, University of Bonn, Bonn, Germany; ^2^Institute of Biomedicine of Seville (IBIS)-Hospital Universitario Virgen del Rocío, CSIC, University of Seville, Seville, Spain; ^3^Department of Biochemistry and Molecular Biology, Faculty of Pharmacy, University of Seville, Seville, Spain

**Keywords:** damaging factors, dopaminergic cell death, early detection, neuroprotection, new treatments

More than 200 years have passed since James Parkinson, in his work *An Essay On The Shaking Palsy*, first gave a unified description of a long-known set of scattered symptoms, and 60 years have passed since the first demonstration of the antiparkinsonian effects of intravenous and orally administered L-DOPA and the insight that the progressive neurodegeneration of dopaminergic neurons in the substantia nigra pars compacta (SNc) underlies the cardinal symptoms of the disease named after James Parkinson (Figure 1). Since then, incredible advances have been made in the understanding of the *Factors contributing to dopaminergic cell death*—the title of this Research Topic. Nevertheless, we have to acknowledge that Parkinson's disease (PD) is still incurable, we do not know how to prevent it, and our essentially only therapeutic approach is the temporary palliative maintenance of dopaminergic signaling.

**Figure 1:**
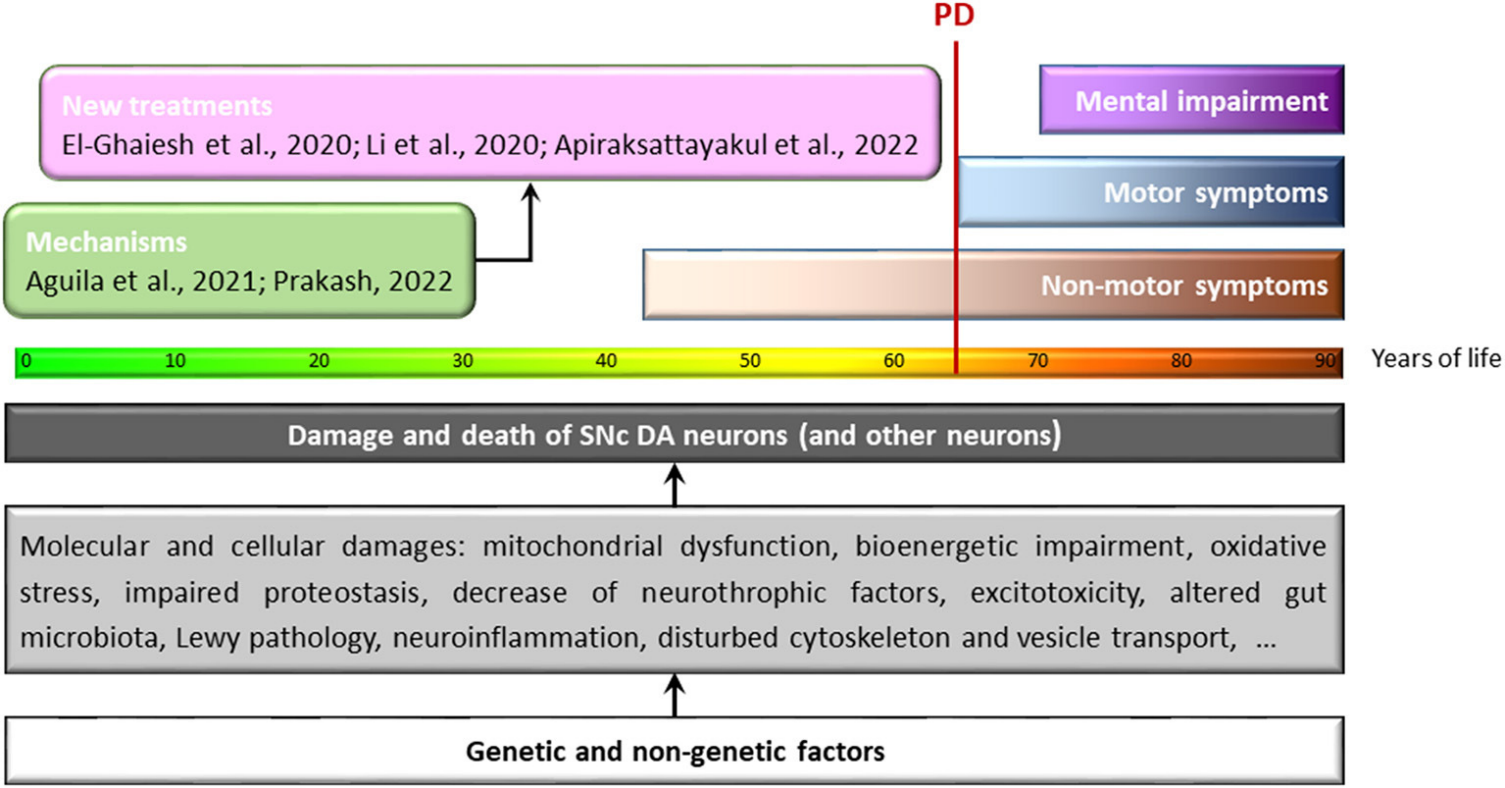
Molecular and cellular damage that can lead to degeneration of SNc-DA neurons during the course of human life. Years of life: the progression of color in the bar represents the loss of functionality associated with the cellular aging process. Below this bar, some of the processes identified as harmful to DA neurons of the SNc (and other neurons) are summarized. These include processes initiated and maintained by genetic and non-genetic factors interacting in complex and hidden ways long before the first symptoms of the disease appear; the end result is the death of these neurons. Unfortunately, when motor symptoms appear—the first unmistakable signs of PD (vertical red line)—neuronal damage is extensive and irreversible. If we are to move beyond the temporary palliative maintenance of dopaminergic signaling that characterizes current treatments, we must identify the asymptomatic death of neurons early, discover the exact mechanisms that cause their death, and then use treatments that can prevent and/or stop neurodegeneration in the prodromal/asymptomatic stages of the disease. This Frontiers Research Topic includes a number of studies addressing either the molecular mechanisms of neurodegeneration (green box) or potential treatment options (pink box).

The objective of this Research Topic was to explore the *Factors contributing to dopaminergic cell death* (Figure 1). It is possible that the compartmentalization of the study of different neurodegenerative diseases has led us to ignore some shared molecular mechanisms. For example, glycogen synthase kinase-3 (GSK-3), well-known for its role in Alzheimer's disease (AD), has emerged only in recent years as a factor to consider in the etiology of PD. The non-competitive GSK-3 tiadiazolidinone inhibitor Tideglusib, already tested in other neurological disorders, was evaluated in this Research Topic by Li et al., showing neuroprotective effects on dopaminergic neurons in an MPTP murine model of PD.

It is necessary to find new therapeutic approaches that help prevent the disease. This objective could be achieved using compounds such as metformin, which have already demonstrated their usefulness in the treatment of other diseases and may be an effective antioxidant and neuroprotective agent (El-Ghaiesh et al.). Furthermore, 17 new bis-sulfonamide derivatives have shown their potential to reduce oxidative stress, restore mitochondrial function and neuronal viability, and enhance the activity of NAD-dependent deacetylase sirtuin-1 (Apiraksattayakul et al.).

To better understand the mechanisms contributing to dopaminergic cell death in PD, the cellular and molecular mechanisms of dopaminergic neurodegeneration are being investigated at increasingly detailed levels. A comparison of the neighboring dopaminergic populations of the SNc and the ventral tegmental area (VTA) appears particularly promising, as the dopaminergic neurons of the VTA are less susceptible to neurodegenerative processes. RNA sequencing of laser capture microdissected human dopaminergic SNc and VTA neurons from healthy and PD patient's brains has uncovered a set of differentially expressed genes that are unique to either SNc or VTA dopaminergic neurons as well as genes that are dysregulated in the SNc of PD patient brains and could thus serve as biomarkers (Aguila et al.).

One of the most interesting and recent plot twists in PD research is that some of the factors that determine neuronal fate in our old age originate early in life, i.e., in the embryonic development of dopaminergic neurons in the midbrain. Prakash summarizes recent findings on the molecular factors involved in dopaminergic neuron development and how they influence their survival in the adult and aging brain.

Identification of factors capable of exerting neuroprotective functions in a damaged brain or an aged brain could increase the survival of dopaminergic neurons and eventually enable a whole new range of treatments more ambitious than current palliative approaches and capable of halting neuronal loss. A cure for Parkinson's disease could then be within reach.

## Author contributions

Both authors listed have made a substantial, direct, and intellectual contribution to the work and approved it for publication.

